# A Single Active-Site Water Molecule Encodes Two Distinct Proton-Coupled Electron Transfer Pathways in Fatty Acid Photodecarboxylase

**DOI:** 10.1021/acsomega.6c06141

**Published:** 2026-10-21

**Authors:** Gianluca Dell’Orletta, Dongping Zhong, Massimiliano Aschi, Isabella Daidone

**Affiliations:** † Department of Physical and Chemical Sciences, 201850 University of L’aquila, via Vetoio (Coppito 1), L’Aquila 67100, Italy; ‡ Center for Ultrafast Science and Technology, School of Chemistry and Chemical Engineering, 2647 Shanghai Jiao Tong University, Shanghai 200240, China

## Abstract

Water molecules are key actors in enzymatic proton-coupled electron transfer (PCET), yet their mechanistic versatility remains underexplored. Using a multiscale computational approach, we show that a previously unidentified water molecule (Wat3) in fatty acid photodecarboxylase (FAP), a natural photoenzyme converting fatty acids into alkanes, mediates radical quenching via two PCET pathways: in the dominant one, which qualitatively agrees with the 100 ns experimental time scale, Wat3 stabilizes the transition state for direct proton transfer from Cys432 to the alkyl radical; in the alternative, the same molecule acts as a proton shuttle in a quasi-concerted two-proton transfer, with the flavin semiquinone providing the electron in both cases. The minor pathway, though less accessible in wild-type FAP, may become operative in engineered variants or related enzymes. Additionally, CO_2_ reacts with the thiolate to yield a thiocarbonate adduct, explaining unassigned crystallographic electron density. These results show how a single water molecule encodes mechanistic diversity in enzymatic PCET.

## Graphical abstract

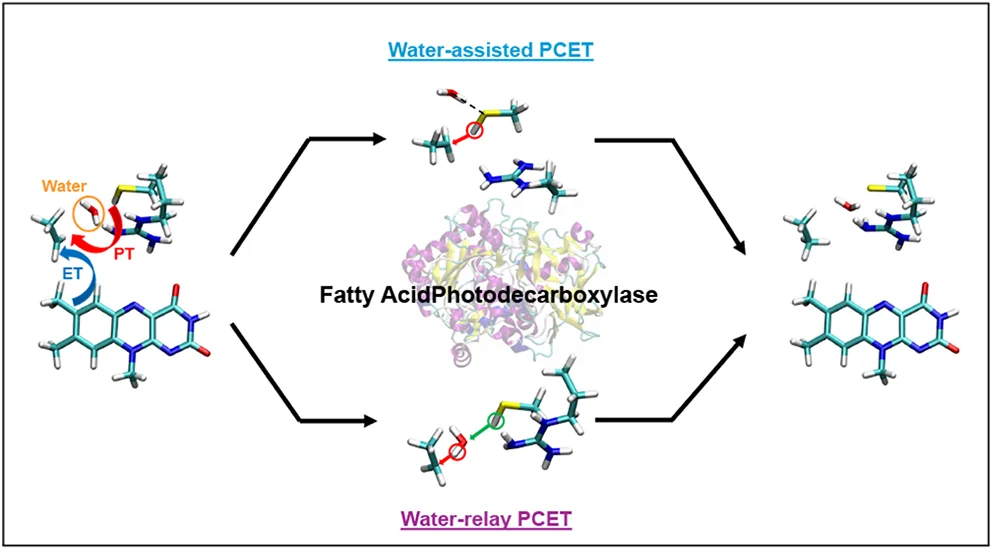

## Introduction

Active-site water molecules are widely recognized as direct contributors to enzymatic catalysis.^1^ In proton-coupled electron transfer (PCET) reactions, which underpin a broad range of enzymatic processes including radical catalysis, oxidoreduction, and energy transduction,^2–5^ water can serve multiple distinct roles: as a hydrogen-bond partner that preorganizes the active site and stabilizes the transition state, as a direct proton donor or acceptor, or as a proton-relay shuttle enabling multiproton transfer events.^6–8^ These roles are not mutually exclusive, and the idea that a single active-site water molecule might support more than one catalytic function, for example adopting different mechanistic modes depending on the conformational state of the enzyme, has rarely been addressed explicitly.

Radical catalysis provides an ideal framework to investigate such behavior, as the efficiency and selectivity of these reactions depend critically on the precise timing and geometry of proton and electron transfer. Photoenzymes, which generate radical intermediates under light control, are particularly well suited for dissecting these mechanisms. To date, only three natural photoenzymes have been identified: DNA photolyases, which harness light energy to repair ultraviolet-induced DNA lesions;^11,12^ light-dependent protochlorophyllide oxidoreductase (LPOR), which catalyzes the light-driven reduction of protochlorophyllide to chlorophyllide in chlorophyll biosynthesis;^13,14^ and fatty acid photodecarboxylase (FAP), which performs the redox-neutral decarboxylation of fatty acids to yield C1-shortened hydrocarbons.^9,15,16^

FAP was recently discovered in the microalga *Chlorella variabilis* (*Cv*FAP) and belongs to the glucose-methanol-choline oxidoreductase family.^15^ Its activity is triggered by photoexcitation of a noncovalently bound flavin adenine dinucleotide (FAD) cofactor within the active site.^9,15^ *Cv*FAP shows a marked preference for mid- to long-chain fatty acids, with highest efficiency for C16–C17 fatty acids.^15^ *Cv*FAP has shown promising potential in the production of chemicals, cosmetics, and biofuels.^17–20^ Several studies have aimed to rationalize the complex catalytic mechanism of *Cv*FAP.^9,10,15,16,21–26^

In a comprehensive study, Sorigué et al.^9^ combined crystallographic, spectroscopic, and computational approaches to propose the catalytic cycle of *Cv*FAP (Figure 1). Upon blue-light excitation, the FAD cofactor reaches its singlet excited state (^1^FAD*), which is quenched within ∼300 ps via forward electron transfer (fET) from the bound fatty acid (R-COO^–^), generating a flavin semiquinone (FAD^•–^) and a fatty-acid radical (R-COO^•^).^9,16^ Subsequent decarboxylation yields an alkyl radical (R^•^) and CO_2_. The catalytic cycle is proposed to proceed either through proton-coupled electron transfer (PCET) or via hydrogen atom transfer (HAT), ultimately affording the alkane product. Reoxidation of FAD^•–^ gives rise to a red-shifted flavin species (FAD_RS_), which relaxes back to the ground state on the millisecond time scale.^9^

**Figure 1:**
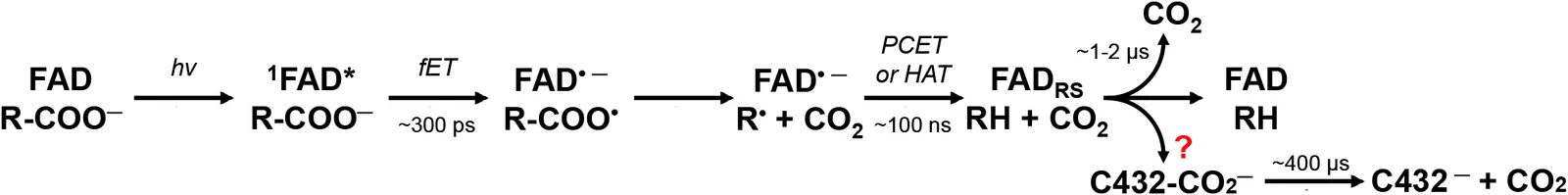
Schematic representation of the *Cv*FAP photocatalytic cycle at room temperature, showing photoinduced, fET-driven decarboxylation, followed by radical quenching via PCET or HAT, leading to the formation of the red-shifted oxidized flavin species FAD_RS_. After this step, the generated CO_2_ is either released to the bulk or is converted into an intermediate species within the active site prior to release.^9,10^

Cryogenic experiments suggested that the transferred proton in the PCET pathway originates from a nearby water molecule (denoted Wat2 in the crystallographic structure),^9^ whereas HAT would involve the hydrogen atom of C432 side chain.^9^ However, infrared spectroscopy of *Cv*FAP in the FAD_RS_ provided no evidence for HAT at cryogenic temperatures, instead pointing toward an arginine-assisted PCET mechanism.^9^ At room temperature, however, the mechanism remains unclear. Concerning the fate of the CO_2_ coproduct, infrared experiments at cryogenic temperatures indicated that CO_2_ could be converted into bicarbonate.^9^

At physiological conditions, however, no direct experimental evidence has yet established the precise mechanism of the PCET (or possibly HAT) step. In contrast, the fate of the CO_2_ coproduct has been investigated using a combination of Fourier-transform infrared and time-resolved infrared spectroscopies, membrane-inlet mass spectrometry, site-directed mutagenesis, isotope-exchange experiments, and computational approaches.^10^ These studies demonstrated that bicarbonate does not form under ambient conditions: approximately 25% of the CO_2_ is directly released into the bulk solvent (in 1–2 μs), while the remaining fraction populates an unidentified transient intermediate that retains CO_2_ in the active site prior to release on time scale roughly estimated to be ∼400 μs. This room-temperature mechanistic model highlights the need to identify the actual CO_2_ intermediate. Based on positive electron density near C432 in room-temperature Time-Resolved Serial Femtosecond Crystallography (TR-SFX) experiments,^9^ Bonvalet et al. proposed a transient sulfur-bound CO_2_ adduct.^10^ In solution, thiolates readily react with CO_2_ to form thiocarbonates $\left({\mathrm{RSCO}}_{2}^{-}\right)$ ;^27,28^ by analogy, a similar adduct may be transiently stabilized in FAP at ambient temperature, where bicarbonate is not detected.^10^

Despite these advances, several key questions remain unresolved under ambient conditions. In particular, the mechanism of the hydrocarbon formation step is still unclear. Could alternative proton-transfer pathways or water molecules contribute beyond the proposed Wat2-mediated mechanism? Is the R451-assisted proton relay required, or might a more direct PCET or HAT pathway operate? Moreover, the identity of the intermediate formed after decarboxylation at room temperature remains unknown. More broadly, these questions raise whether a single active-site water molecule can support distinct PCET mechanisms with different energetic barriers, and how the operative pathway is selected by the enzyme conformational ensemble. Here, we address these issues using a multiscale computational approach combining molecular dynamics (MD), quantum mechanical/molecular mechanical (QM/MM) calculations, and the Perturbed Matrix Method (PMM),^29–32^ with *Cv*FAP serving as paradigmatic model system for photoenzymatic catalysis.

## Results and Discussion

In our previous studies,^24,25^ we reported extensive MD simulations of *Cv*FAP, with an aggregate sampling time on the microsecond time scale at room temperature. Based on these simulations, we computed the FAD absorption spectrum, which showed very good agreement with experiment, supporting the reliability of the sampled conformational ensemble. In the present work, we build upon one representative trajectory from this data set to investigate the hydration pattern in the vicinity of the substrate with the aim of identifying water molecules that may play a functional role in the subsequent proton–electron transfer step leading to alkane formation. We refer to this simulation as the dark-state MD simulation.

For each frame, we identified the water molecule closest to the substrate’s C_α_ atom, i.e., the atom that, in its radical form after decarboxylation, is expected to receive the proton. We classified a water molecule as Wat2 when it occupied the corresponding crystallographic pocket, defined by two geometric criteria: a distance ≤ 3.5 Å from both the side-chain nitrogen of Q486 and the backbone oxygen of S573. We classified the closest water molecule as Wat3 when it was within 3.5 Å of the T430 side-chain oxygen and within 4.0 Å of the sulfur atom of C432. The locations of the Wat2 and Wat3 sites are illustrated in Figure 2A. We then monitored the distance between the oxygen atom of the closest water molecule and the substrate’s C_α_ atom (Figure 2B and Figure S1 in the Supporting Information, SI). Note that we selected MD simulations in the dark-state ensemble rather than the radical-state ensemble because force-field parameters for radical intermediates are less reliable over long time scales. Wat2 was the closest water molecule in only ∼10% of the MD frames, whereas Wat3 was the closest in ∼74% of the frames. Thus, the water molecule closest to C_α_ is most frequently located at the Wat3 site, indicating that Wat3 represents a highly populated hydration site.

**Figure 2:**
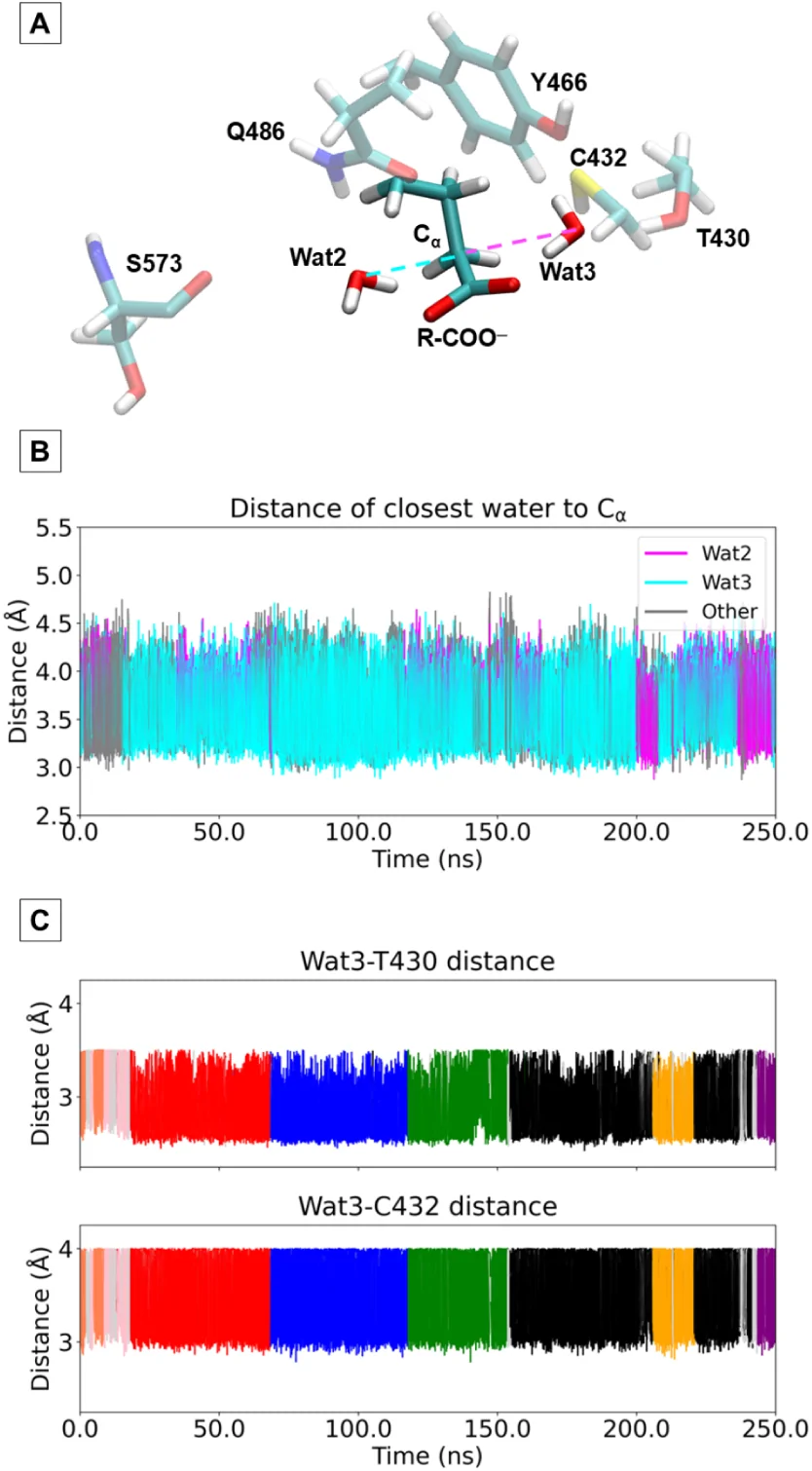
A) Representative structure extracted along the dark-state MD simulation highlighting the water molecules, Wat2 and Wat3, closest to the reactive C_α_ carbon of the R-COO^–^ substrate. The amino acid residues T430, C432, Y466, Q486, and S573 are also shown. B) Time evolution of the distances between the C_α_ atom of R-COO^–^ and the oxygen atom of the closest water molecule. The trajectory of Wat2 is shown in magenta (10%), that of Wat3 in cyan (74%), while all other water molecules are shown in gray (16%). C) Time evolution of the distances between the oxygen atom of Wat3 and the T430 side-chain oxygen and the sulfur atom of C432 along the MD simulation. Different colors indicate distinct water molecules occupying the Wat3 site. The two distances are reported for all frames in which a Wat3 water molecule is present at this site, corresponding to ∼86% of the MD trajectory.

To assess the persistence of Wat3 occupancy, we monitored its distances to the oxygen atom of T430 and the sulfur atom of C432 throughout the MD trajectory. Distinct colors in Figure 2C correspond to different water molecules transiently populating this position. The average residence time of each water molecule is on the order of tens of nanoseconds, indicating that this hydration site is dynamically stable on the time scale of the early photochemical events. A similar dynamic behavior was observed for Wat2, as shown in Figure S1 in the SI. Overall, Wat3 remains systematically closer to the reactive C_α_ position than Wat2 (see Figure S1 in the SI). Although no water molecule is resolved at the Wat3 position in the dark-state room-temperature serial femtosecond crystallography structure (PDB 6ZH7), this site lies within a region where TR-SFX experiments detected light-induced changes in electron density,^9^ supporting a dynamic hydration environment. Our previous MD simulations showed that the cavity adjacent to C432 became populated by a water molecule already during equilibration, prior to the production simulations.^24^ Thus, Wat3 may represent a dynamically populated hydration site that is not captured in a single dark-state structure, and a plausible candidate for involvement in the PT/ET step.

Inspired by the TR-SFX results^9^ and by the proposed direct involvement of C432 in the PT/ET process during the subsequent hydrocarbon formation step,^21^ we monitored key structural descriptors of C432 along the MD trajectory, focusing on the subset of configurations in which Wat3 is present. Specifically, we analyzed the distance between the sulfur atom of C432 and the C_α_ atom of the alkyl radical (S_C432_–C_α_) and the S_C432_–H_C432_···C_α_ angle defined by the sulfur and hydrogen atoms of C432 and the C_α_ atom. The S_C432_–C_α_/S_C432_–H_C432_···C_α_ two-dimensional distribution (reported in Figure 3) reveals the presence of two C432 basins. In both cases, the most populated S_C432_–C_α_ distance is approximately 6 Å. The angular distributions, however, differ markedly between the two regions. In the dominant basin (C432-out basin), the S_C432_–H_C432_···C_α_ angle spans the 20–100° range, corresponding to configurations in which the proton is oriented away from the C_α_ atom. In the less populated basin (C432-in basin), the angle falls within the 120–180° range, indicative of geometries in which the proton is directed toward the C_α_ atom. These differences are reflected in the hydrogen-bonding network of the two basins. In the C432-out basin, T430 donates to Wat3, which in turn donates to the substrate carboxylate (R–COO^–^) and to Y466. In the C432-in basin, this network is preserved, with the additional involvement of C432 as a hydrogen-bond donor to Wat3.

**Figure 3:**
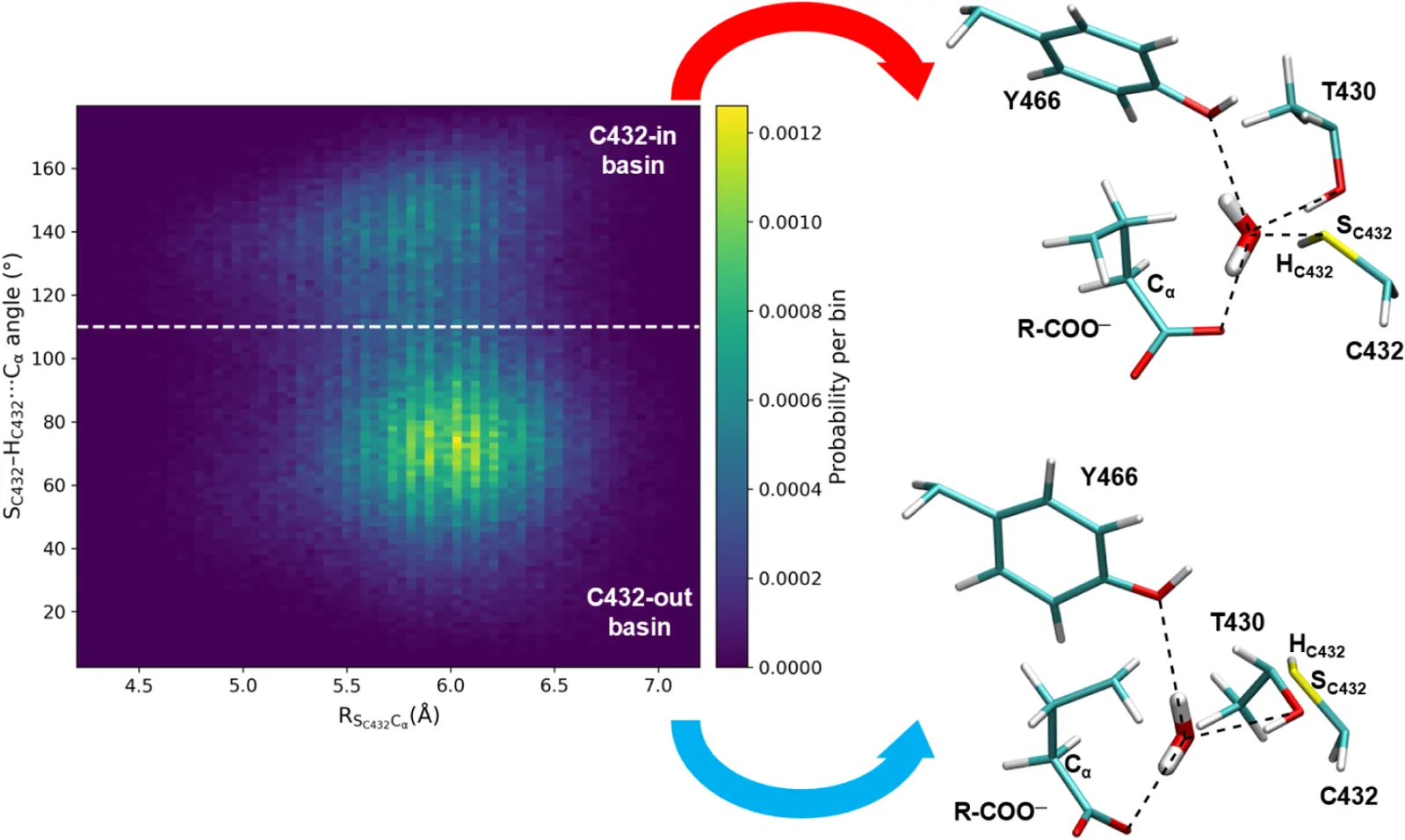
Two-dimensional probability distribution of the distance between the sulfur atom of C432 and the C_α_ atom of the alkyl radical (S_C432_–C_α_) and of the S_C432_–H_C432_···C_α_ angle defined by the sulfur and hydrogen atoms of C432 and the C_α_ atom, sampled along the dark-state MD simulation. The distribution reveals two basins: a highly populated region in which C432 points away from C_α_ (C432-out basin) and a less populated region in which C432 points toward C_α_ (C432-in basin). Representative structures highlighting the network of hydrogen bonds for each basin are also shown.

A representative structure was extracted from each basin, and from each a QM/MM model of the initial radical state was constructed as described in the QM/MM Calculations section, yielding the C15 alkyl radical and CO_2_. In the geometry derived from the C432-in basin, the S–H group of C432 donates a hydrogen bond to Wat3 (Figures 3, 4A, D, and S2); in the other, C432 acts as a hydrogen-bond acceptor from Wat3 (Figure 3 and S3). Starting from each geometry, two relaxed QM/MM scans were performed to probe two mechanistic scenarios: either a direct PCET (or possibly a HAT) from C432 to the radical alkyl or a water-mediated PCET relay involving Wat3. To this end, the first scan was calculated along the distance between the C_α_ radical carbon of the C15 alkyl radical and the hydrogen atom of C432 (H_C432_), the second along the distance between C_α_ and the hydrogen atom of Wat3 undergoing transfer (H_w_). Calculations were carried out on the unrestricted singlet surface to properly describe the open-shell species involved in the reaction. The resulting QM/MM energy profiles are reported in Figure S4 of the SI. The scenario is similar for both starting structures, but the transition energies are consistently higher for the geometry started from the C432-out basin. We thus performed further detailed analyses only for the geometry derived from the C432-in basin.

**Figure 4:**
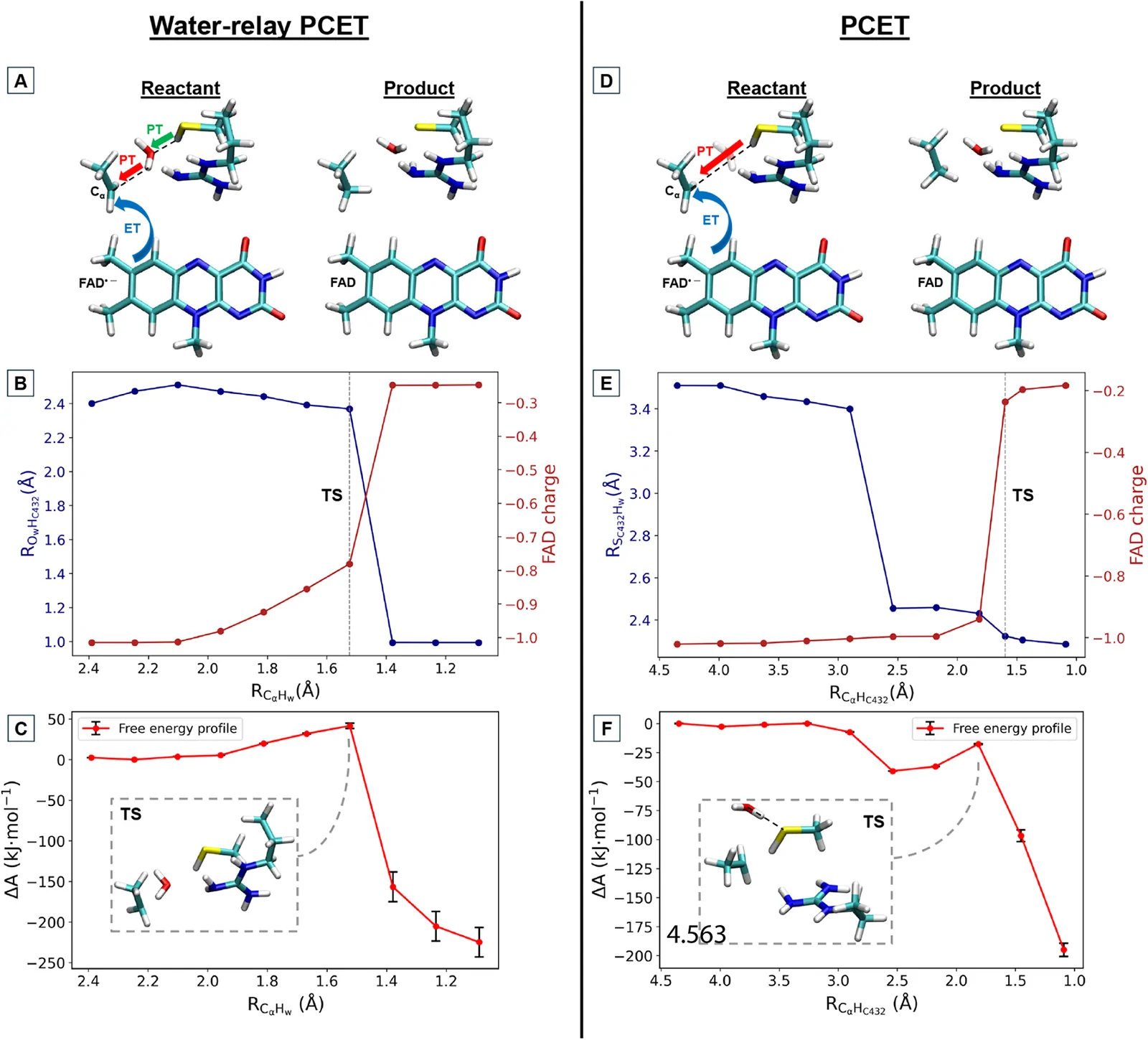
A) Structures of the QM region corresponding to the reactant and product states of the water-relay PCET step. The proton-transfer coordinate $\left(R_{C_{\alpha }H_{w}}\right)$ used in the QM/MM scan is highlighted in red in the reactant structure, while the green arrow indicates the direction of proton transfer from C432 to the water molecule. B) Evolution of the distance between the oxygen atom of Wat3, O_w_, and the proton of C432, H_C432_, (dark blue), together with the CHELPG-derived charge on FAD (dark red), plotted as a function of the proton-transfer coordinate $R_{C_{\alpha }H_{w}}$ used in the QM/MM scan. C) PMM-derived free-energy profile (red) for the PCET reaction as a function of the proton-transfer coordinate $R_{C_{\alpha }H_{w}}$ . The transition state (i.e., the highest-energy point along the profile) is shown in the inset and corresponds to an activation free energy of Δ*A* ^‡^ = 41.5 ± 3.2 kJ·mol^–1^. D) Structures of the QM region corresponding to the reactant and product states of the direct PCET step involving the C432 side chain. The proton-transfer coordinate $\left(R_{C_{\alpha }H_{C432}}\right)$ used in the QM/MM scan is highlighted in red in the reactant structure. E) Evolution of the distance between the sulfur atom of C432, S_C432_, and the hydrogen atom of Wat3, H_w_ (dark blue), together with the CHELPG-derived charge on FAD (dark red), plotted as a function of the proton-transfer coordinate $R_{C_{\alpha }H_{C432}}$ used in the QM/MM scan. F) PMM-derived free-energy profile (red) for the PCET reaction as a function of the proton-transfer coordinate $R_{C_{\alpha }H_{C432}}$ . The transition state (i.e., the highest-energy point along the profile) is shown in the inset and corresponds to an activation free energy of Δ*A* ^‡^ = 23.3 ± 0.4 kJ·mol^–1^. Error bars in the free-energy profiles represent the uncertainty at each point, estimated as half of the difference between the values obtained from two 100 ns MD simulations.

Notably, in the water-relay scenario, the PT/ET event is accompanied by a slightly asynchronous second proton transfer from C432 to Wat3 (note that no constraint was applied on this proton distance, $R_{O_{w}H_{C432}}$ ). In this mechanism, Wat3 donates a proton to the alkyl radical while being reprotonated by C432, ultimately leading to a deprotonated cysteine and alkane formation via ET from FAD^•–^ (see Figure 4A). Changes in electron density were monitored through CHELPG partial charges,^33^ showing that the unpaired electron shifts from FAD^•–^ to the C15 radical upon crossing the transition state region (Figure 4B). This behavior supports a quasi-synchronous electron–proton transfer, with electron relocation occurring almost concomitantly with the proton-transfer steps. We refer to this mechanism as the water-relay PCET (wr-PCET).

Also in the direct pathway, the electron is donated by the semiquinone FAD, as evidenced by the evolution of the partial charges on the FAD moiety along the proton-transfer coordinate ( $R_{C_{\alpha }H_{C432}}$ , Figure 4E). Upon crossing the transition state region, the unpaired electron density is largely transferred from FAD^•–^ to the C15 radical. Regarding proton motion, following an initial approach of the cysteine toward the alkyl radical, the proton is ultimately transferred to the radical center. Interestingly, reaching the TS is accompanied by the formation of a hydrogen bond donated by Wat3 to the sulfur atom (see Figure 4E). This behavior is consistent with a quasi-synchronous PCET, in which electron relocation occurs nearly concomitantly with proton transfer. We designate this mechanism as the water-assisted PCET pathway.

Given the key role of hydrogen bonding in PCET processes,^4,5^ we analyzed the hydrogen-bonding network along the two reaction pathways, as illustrated in Figure 5 and reported in Table S1. In the water-relay PCET mechanism, the Wat3-centered hydrogen-bond network involving T430, C432, and Y466 is largely preserved along the reaction coordinate. The SH group of C432 initially donates a hydrogen bond to Wat3; upon approaching the transition state, Wat3 transfers one of its own protons to the alkyl radical while maintaining a hydrogen-bond interaction with C432. In the product state, the proton from C432 is transferred to Wat3, which in turn forms a hydrogen bond with the deprotonated C432, maintaining the connectivity of the network. In contrast, in the direct PCET pathway, C432 initially acts as a hydrogen-bond donor to Wat3 in the reactant state, but a rearrangement occurs upon approaching the transition state, leading Wat3 to switch its role and donate a hydrogen bond to the sulfur atom of C432. This interaction is retained in the product state. Notably, the hydrogen bonds between Wat3 and both T430 and Y466 remain largely conserved throughout the scan.

**Figure 5:**
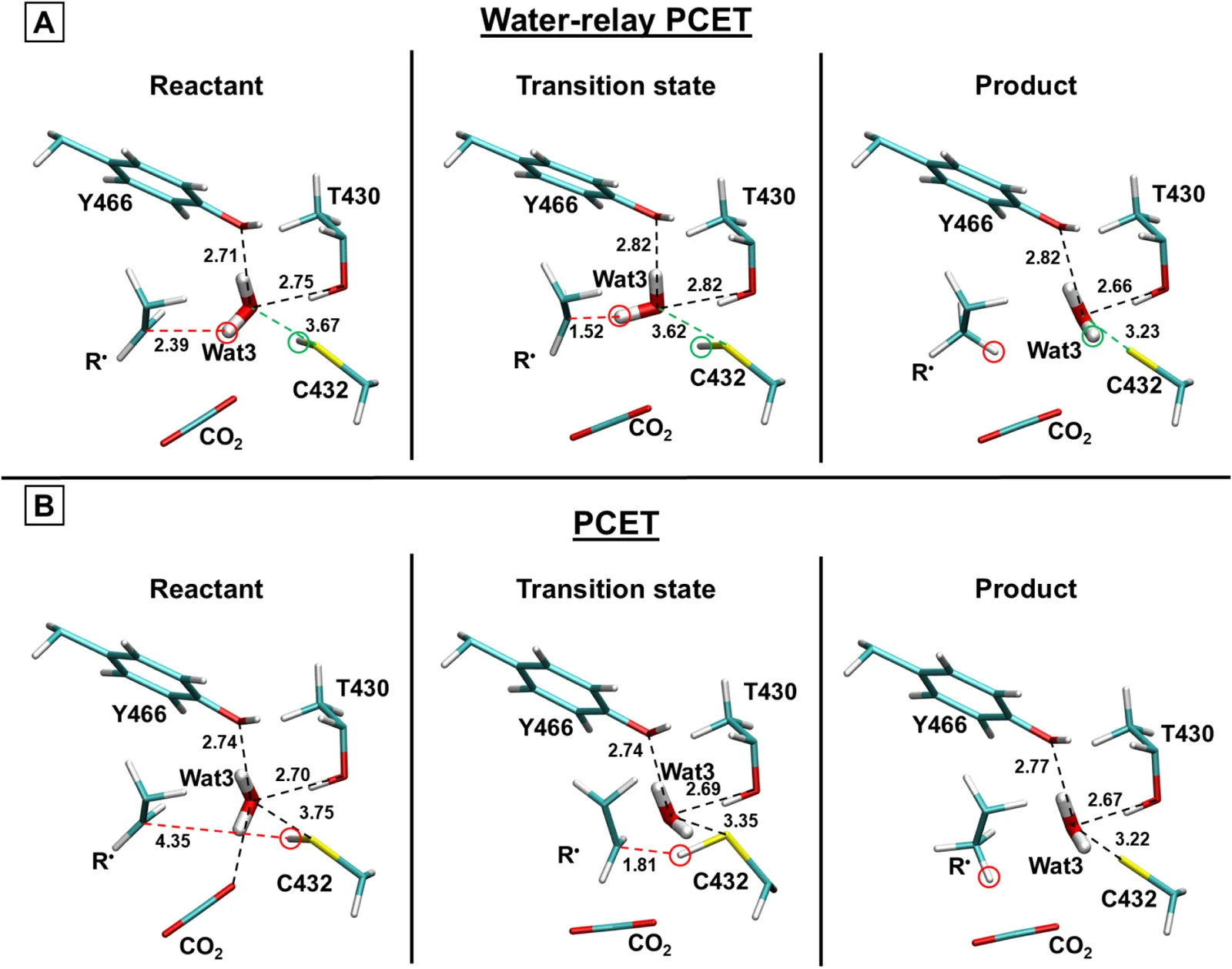
Hydrogen bonds in the reactant, transition state, and product for the water-relay PCET and direct PCET pathways obtained from scans starting from the C432-in basin. The distance defining the reaction coordinate is shown in red.

The structures obtained along the scan were subsequently used to evaluate the corresponding free-energy profile within the PMM framework^29,31,34,35^ (see the PMM for Reaction Free Energy Calculation Section for details). The physical advantage of the PMM approach is that it accounts for the fluctuating electrostatic environment provided by the protein and solvent, allowing the reaction free-energy profile to be evaluated over an ensemble of MD configurations rather than for a single static structure (Figure 4C and F). This analysis yielded an activation free energy of Δ*A* ^‡^ = 41.5 ± 3.2 kJ·mol^–1^ for the wr-PCET mechanism and Δ*A* ^‡^ = 23.3 ± 0.4 kJ·mol^–1^ for the water-assisted PCET mechanism, thus indicating that the direct, water-assisted PCET is favored and will thus dominate the observed kinetics. In order to obtain a rough estimate of the rate constant of the process, we employed the Eyring model.^36^ Within this framework, the computed barrier corresponds to a catalytic rate constant *k*_*cat*_ of (1.8 ns)^−1^. Considering the probability of sampling the reactive state (i.e., the C432-in basin described above) of 0.25, the population-averaged (observed) PCET rate *k*_*obs*_ becomes (7.3 ns)^−1^. The probability associated with the starting state was estimated as ∼0.74 × 0.34 = 0.25, where 0.74 is the population of structures with Wat3 present and 0.34 is the fraction of configurations belonging to the C432-in basin from which the structure was extracted. This time constant is faster than the ∼100 ns measured experimentally, a discrepancy attributable to the inherent approximations of the Eyring framework and the PMM approach; nevertheless, it correctly captures the nanosecond time scale measured experimentally.

Sampling limitations associated with the choice of the reactive starting structure were addressed by performing additional scans initiated from a second configuration extracted from the C432-out conformational basin, as mentioned above. These independent calculations revealed the same PCET mechanisms, although with higher activation energies, supporting the robustness of the mechanistic picture. The PCET calculations also involve a number of methodological approximations, including the classical treatment of the transferring proton along a one-dimensional reaction coordinate, the neglect of vibronic excited states, and the intrinsic limitations of the employed DFT methods and classical MD force fields. While these aspects may influence the absolute quantitative values of the computed free-energy barriers, the resulting free-energy profiles provide a consistent and chemically meaningful description of the process. Notably, the experimentally measured 100 ns time scale of the PCET step suggests that the reaction proceeds in a regime where nuclear tunneling and vibronic coupling effects are not expected to dominate, lending support to the present treatment.^9^ More advanced theoretical frameworks that explicitly account for such effects have been reported and applied to a variety of PCET processes in proteins.^37–41^

According to a hypothesis put forward by Bonvalet et al.,^10^ the CO_2_ species generated upon decarboxylation can follow two possible pathways at room temperature. A fraction of the population is released into the bulk solvent, whereas the majority forms a transient intermediate prior to escape. One possibility is that the newly formed C432^–^ thiolate participates in this intermediate process. To explore this hypothesis, we performed several QM/MM scans along the distance between the sulfur atom of the thiolate and the carbon atom of CO_2_ (see the QM/MM Calculations section for details of the starting geometries and Figure S5). The resulting energy profiles (Figure 6A) reveal the formation of a thiocarbonate adduct (Figure 6B) that is more stable than the reactant state and separated from it by only a small activation barrier (around 20 kJ·mol^–1^). This sulfur-bound intermediate is consistent with the positive electron density observed in the vicinity of C432 in room-temperature TR-SFX experiments^9^ (see Figure 6C).

**Figure 6:**
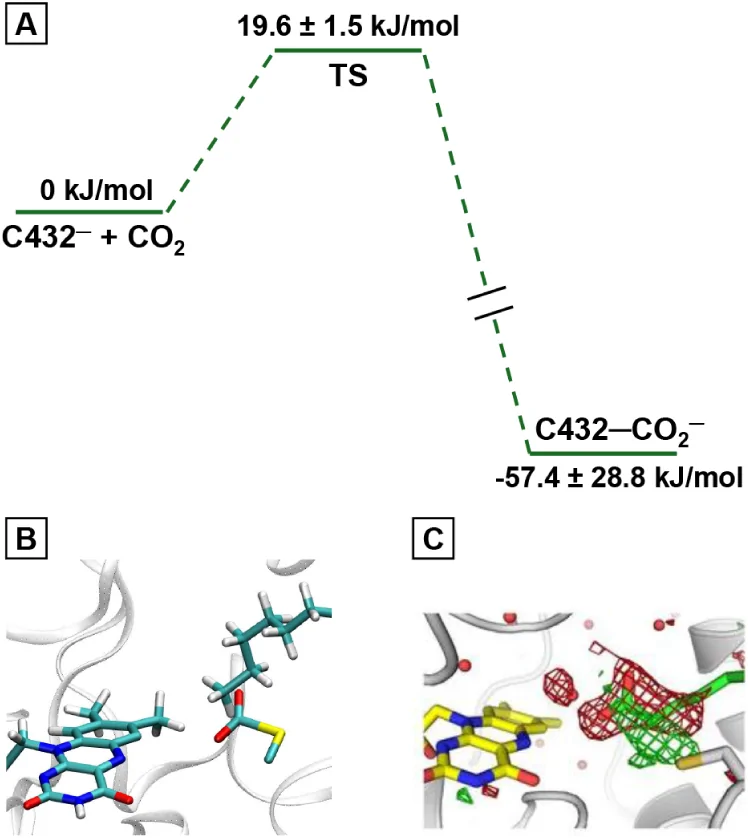
A) QM/MM energy profiles for thiocarbonate formation obtained by averaging over four independent profiles. Reported ΔE values (kJ·mol^–1^) correspond to the transition state and product, with error bars representing the uncertainty estimated from the four profiles. B) Representative thiocarbonate structure formed along the QM/MM scans. C) Light-induced difference electron density map calculated at 300 ns in the TR-SFX experiment, revealing a difference electron density feature in proximity to C432 consistent with thiocarbonate formation. Reproduced from Figure 5B in ref 7. D. Sorigué, K. Hadjidemetriou, S. Blangy et al., Science 2021, 372, eabd5687), with permission from AAAS.

## Conclusions

By means of a comprehensive multiscale computational analysis, we establish a coherent and experimentally consistent mechanism for the photocatalytic activity of *Cv*FAP at ambient temperature. We identify a previously unrecognized water molecule (Wat3) that plays a central role in the alkane-forming step, bridging cysteine C432 and the alkyl radical. Its position is consistent with the light-induced electron density changes observed crystallographically near C432 and T430, supporting its functional relevance. Our results indicate that catalysis proceeds via a proton-coupled electron transfer (PCET) mechanism, in which the electron is donated by the semiquinone flavin intermediate (FAD^•–^) and the proton by C432, assisted by Wat3 through hydrogen-bonding. The involvement of a structured water molecule underscores the functional importance of the active-site hydrogen-bond network in orchestrating spatially and temporally controlled proton–electron transfer, as also highlighted for other radical-relay enzymes.^8^ This mechanism is consistent with the proposal by Heyes et al.,^21^ who identified C432 as a key actor in the reductive step, but differs in that they hypothesized a concerted hydrogen atom transfer (HAT) directly from C432. Moreover, our model resolves a structural inconsistency: Wat2, previously proposed to mediate PCET at cryogenic conditions,^9^ lies on the opposite side of the active site relative to C432 and is poorly positioned to account for the room-temperature electron density changes. In contrast, Wat3 establishes a well-defined hydrogen-bond network linking C432 to the substrate radical, providing a geometrically and chemically consistent description of the ambient-temperature mechanism.

A key result of this study is the identification, within the same active site, of two mechanistically distinct roles for Wat3. In the kinetically dominant pathway (direct PCET, Δ*A* ^‡^ ≈ 23 kJ·mol^–1^), Wat3 acts as a hydrogen-bond donor that stabilizes the transition state without being itself the proton carrier. In the alternative pathway (wr-PCET, Δ*A* ^‡^ ≈ 42 kJ·mol^–1^), the same water molecule functions as a proton-relay shuttle, mediating a quasi-concerted two-proton transfer event. While the higher barrier of the relay pathway renders it kinetically negligible in *Cv*FAP under ambient conditions, this route remains energetically accessible and may become operative in enzyme variants or related photoenzymes where active-site geometry disfavors the direct assisted pathway. The ability of a single water molecule to encode two distinct PCET mechanisms within the same binding site represents a form of catalytic versatility that has so far been largely overlooked, at least in FAP. Mechanistically related scenarios, in which water molecules or polar residues mediate proton transfer concurrently with electron transfer in radical enzymes, have been discussed in ribonucleotide reductase,^7,8^ cytochrome *c* oxidase,^6^ and complex I,^42^ but the explicit competition between a water-assisted and a water-relay PCET mode at the same site has not been previously characterized.

We further show that, after PCET, CO_2_ can react with the newly formed cysteine thiolate to form a transient thiocarbonate adduct via a low energy barrier. This intermediate provides a plausible explanation for the additional electron density observed near C432 in the SFX experiments, as was recently suggested.^9,10^ Our calculations thus offer a direct structural interpretation of the previously unidentified CO_2_-derived species.

Finally, we summarize the mechanisms proposed to operate at cryogenic and ambient temperatures to provide a comprehensive scheme. The overview shown in Figure 7 is intended to assist the reader in rationalizing the current understanding of the *Cv*FAP PCET step and the fate of CO_2_ under different temperature conditions. At ambient temperature, we compare the R451-mediated PCET pathway recently proposed computationally,^26^ with the C432/Wat3-mediated pathways identified in the present study. These mechanisms are considered alongside the pathways previously proposed under cryogenic conditions.^9^

**Figure 7:**
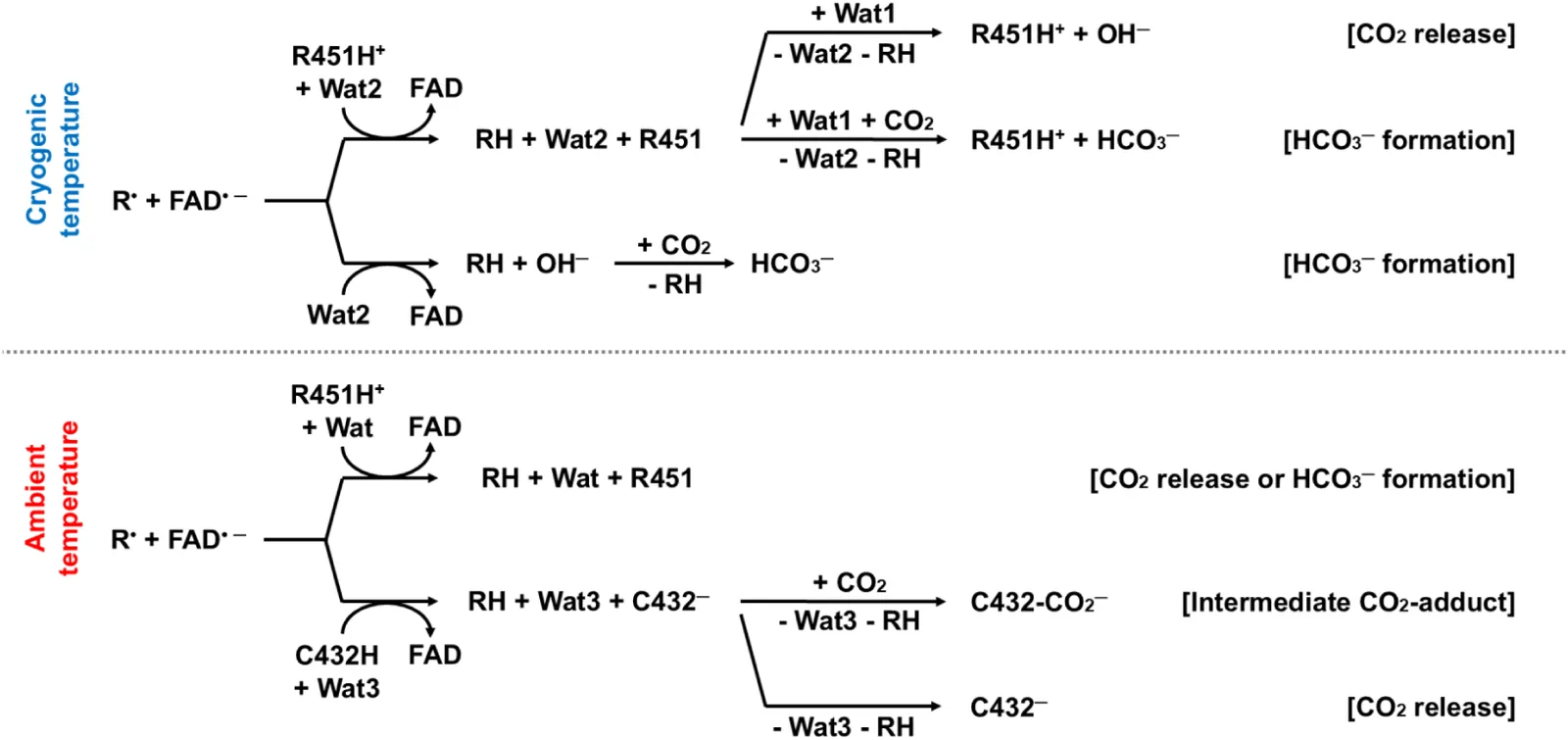
Comprehensive mechanistic model integrating the insights obtained here under ambient conditions with the pathways previously proposed at cryogenic temperatures.^9^ At room temperature, the first pathway corresponds to the R451-mediated PCET mechanism recently proposed computationally,^26^ in which proton transfer from R451 to the alkyl radical is mediated by an active-site water molecule. The pathways identified in the present study instead involve C432 and Wat3: in the water-assisted PCET pathway, Wat3 facilitates proton transfer from C432 during electron transfer from FAD^•–^, whereas in the water-relay PCET pathway, Wat3 acts as a proton shuttle between C432 and the alkyl radical. Following PCET, CO_2_ may either be directly released from the active site or react with the newly formed C432^–^ to yield a sulfur-bound thiocarbonate adduct. At cryogenic temperatures, in contrast, the proposed PCET step involves Wat2. Proton transfer may occur either via direct donation from Wat2 to the alkyl radical, generating the alkane and a hydroxyl species that can lead to bicarbonate formation,^9^ or via Wat2-mediated proton transfer from R451, resulting in transient deprotonation of R451, which is proposed to be reprotonated by Wat1.^9,10^ In this scenario, CO_2_ may react with the newly formed hydroxyl species to form ${\mathrm{HCO}}_{3}^{-}$ .

Our mechanistic model, encompassing both the PCET step and the formation of a transient CO_2_–cysteine adduct, provides a consistent description of the catalytic process at room temperature reconciling key available structural, spectroscopic, and kinetic data.^10^ We note, however, that the present computational framework focuses on pathways involving C432 and Wat3, and does not preclude the possibility that alternative routes may also contribute to the overall reactivity, as was very recently hypothesized.^26^ More broadly, the dual catalytic role uncovered for Wat3, as a transition-state stabilizer in one conformational context and as a proton relay in another, illustrates how a single buried water molecule can encode mechanistic diversity in enzyme active sites. This principle may extend beyond FAP to other radical enzymes, photoenzymes, and engineered biocatalysts where active-site hydration is a tunable design parameter.

## Computational Methods

### Quantum Mechanical/Molecular Mechanics Calculations

All quantum chemical calculations were performed with the ORCA package^43^ at the QM/MM level using the electrostatic embedding scheme. The MM region was described using the Amber ff14SB force field^44^ for the protein, while the General Amber Force Field (GAFF)^45^ was used for all other molecular species. Water molecules were modeled with the TIP3P model.^46^

To investigate the PCET reaction, two starting structures were extracted from the dark-state MD simulation, as described in the Results and Discussion section. Each structure was subjected to a QM/MM geometry optimization in which the C–C distance between the CO_2_ moiety and the α-carbon atom of the fatty acid was constrained to 3 Å in order to promote C–C bond cleavage, thereby generating the C15 alkyl radical and CO_2_. In these preparatory calculations, the QM region included only the C_2_–COO^–^ fragment of the C16 fatty acid (R-COO^–^). The MM active region comprised residues I130, F134, A171, L173, T430, C432, R451, V463, Y466, Q486, S573, S574, N575, G622, the FAD cofactor, the C13 tail of R-COO^–^ within the active site, and all water molecules within 5 Å of the carbon atom of the CO_2_ moiety. The remaining atoms were assigned to the MM inactive region and kept frozen during optimization. The QM region was treated using Density Functional Theory (DFT)^47^ at the B3LYP^48^/def2-TZVP^49^ level, including Grimme’s D3 dispersion correction with Becke–Johnson damping (D3BJ).^50^

During CO2 detachment, the hydrogen-bonding network involving Wat3 remains largely preserved. Wat3 undergoes only a minor rearrangement while maintaining its interactions with the surrounding active-site residues and the oxygen atom of CO2. Therefore, CO2 detachment does not result in a substantial reorientation of the Wat3 hydrogen-bonding network. The optimized radical structures were subsequently used as starting points for the PCET investigation. From each structure, a relaxed QM/MM scan (10 points) was performed along the distance between the radical carbon atom of C15 and the transferring hydrogen atom of Wat3 (a newly identified water molecule discussed in the 0000Results and Discussion section). The number of scan points was chosen to ensure adequate sampling of the reaction coordinate while maintaining computational feasibility. The QM region comprised the side chains of R451 and C432, the isoalloxazine moiety of FAD, the CH_2_–CH_2_ fragment of the C15 radical, and the Wat3 molecule. All PCET scans were carried out at the unrestricted B3LYP-D3BJ/def2-TZVP level of theory,^48–50^ appropriate for the description of the open-shell species involved in the reaction.

For the investigation of thiocarbonate formation, relaxed QM/MM scans (10 points) were performed along the distance between the sulfur atom of C432 and the carbon atom of the CO_2_ molecule, starting from the structures obtained from the PCET scans. In these scans, the QM region included the side chains of C432 and R451 together with the CO_2_ moiety, while the MM active region was identical to that used in the previous QM/MM calculations.

### Perturbed Matrix Method for Reaction Free Energy Calculations

To estimate the reaction free energy, i.e., the variation of the free energy along a reaction coordinate, we employed the Perturbed Matrix Method (PMM) in combination with molecular dynamics (MD) simulations (hereafter MD–PMM).

The MD–PMM methodology has been described in detail elsewhere.^34,35^ Here, only a brief summary is provided. The MD–PMM is a theoretical–computational perturbative approach in which the system is divided into two parts: the *quantum center* (QC), described at the quantum mechanical level, and the surrounding environment, which is treated as a semiclassical atomic–molecular field that electrostatically interacts with the QC. In this work, the QM region used in the QM/MM calculations was considered as the QC, while all remaining atoms constituted the perturbing environment.

This approach allows the use of a large number of atomic configurations of the entire system, as obtained from classical MD simulations, thus ensuring proper sampling of the phase space and enabling reconstruction of the ensemble of perturbed electronic eigenstates of the QC. A set of unperturbed QC eigenstates $\left(\phi_{j}^{0}\right)$ , previously computed at the QM/MM level (see previous section), was used as the basis set to represent the perturbed operator of the QC within the dipolar approximation:^35^

1 $$Ĥ={Ĥ}^{0}+Îq_{T}V+{Ẑ}_{1}$$

2 $${Ẑ}_{1}=-E\cdot \left\langle \phi_{j}^{0}\right|\mathrm{\mu ̂}\left|\phi_{i}^{0}\right\rangle$$

where *Ĥ* ^0^ is the unperturbed Hamiltonian matrix (constructed with the unperturbed QC eigenstates), *Î* is the identity matrix, *q*_*T*_ is the total charge of the QC, V is the electrostatic potential exerted by the environment on the QC center of mass, ***μ̂*** is the electric dipole operator, and **E** is the electric field produced by the environment at the QC center of mass at each MD frame (details of the MD simulations are reported below). $\phi_{i}^{0}$ are the unperturbed electronic eigenstates of the QC. This procedure allows the perturbed electronic energies and dipoles to be evaluated for each frame of the MD trajectory.

By recalculating the basis set $\phi_{i}^{0}$ along a predefined reaction coordinate, we obtained, for each frame of the MD simulation, a collection of perturbed electronic eigenvalues (*U*) as a function of the QC position along the reaction coordinate. The free energy variation Δ*A* along the reaction coordinate was then computed using the following statistical relation:^29,51–53^

3 $$\Delta A\approx -RT\ln\left\langle e^{-\frac{\Delta U}{RT}}\right\rangle$$

where R is the gas constant (8.314·10^–3^ kJ·mol^–1^·K^–1^), T is the temperature (300 K), and Δ*U* is the difference between the perturbed energies at a given point along the reaction coordinate and the reactant state. The angle brackets denote the ensemble average over the MD trajectory. In this work, the calculation was performed using two 100 ns-long MD trajectories. The final reaction free energy Δ*A* was obtained by averaging the results from the two MD trajectories, while the associated uncertainty at each point along the reaction coordinate was estimated as half of the difference between the two values. The computed activation free energy Δ*A* ^‡^ was then employed in the Eyring equation^54^ to estimate the rate constant:

4 $$k_{cat}=\frac{k_{B}T}{h}e^{-\Delta A^{‡}/RT}$$

where *k*_*B*_ is the Boltzmann constant and *h* is Planck’s constant. To account for the pre-equilibrium associated with the presence of Wat3 in the reactive position, we computed the overall rate constant for the PCET reaction using^55^

5 $$k_{obs}=k_{cat}\cdot \frac{K}{1+K}$$

where *k*_*cat*_ is the rate constant obtained from the Eyring equation (eq 4) and *K* is the equilibrium constant corresponding to the probability of sampling Wat3 in the MD trajectory. *K* was calculated as

6 $$K=\frac{P}{1-P}$$

with *P* being the probability of sampling the specific configuration of the system.

#### Molecular Dynamics Simulations

Molecular dynamics simulations to be used in the MD-PMM calculations were performed starting from the structures used as starting points for the QM/MM scans. Each system comprised the protein, the FAD cofactor, the CO_2_ molecule, the C15 alkyl radical, the palmitic acid molecule located outside the active site, and the Wat3 water molecule. Parameters for semiquinone anion FAD cofactor were taken from a recently developed flavin force field.^56^ Parameters for the C15 alkyl radical were derived from those of the corresponding C15 alkane. The parameters for the C15 alkane, the C16 fatty acid, and CO_2_ were obtained using the CHARMM General Force Field (CGenFF) through the official web server.^57,58^

Protein residues were modeled using the CHARMM36 force field,^59^ and solvent molecules were described using the modified TIP3P water model.^46^ As in our previous simulations,^24^ the protonation states of amino acid residues were assigned using PROPKA,^60^ based on the experimental pH of 8.5. In the active site, Y466 was modeled in its neutral state, while R451 was modeled in its protonated state. C432 was modeled in its deprotonated state to represent the state of the residue following proton transfer during the PCET event. Histidine protonation states were assigned by visual inspection of the local chemical environment, considering potential hydrogen-bonding interactions. Counterions were added to neutralize the system. To preserve the structural features of the starting structures of the QM/MM scans, atoms belonging to the original QM region were restrained using harmonic positional restraints with a force constant of 10^5^ kJ mol^–1^ nm^–2^.

All MD simulations were carried out using the GROMACS software package.^61^ Each system was placed in a dodecahedral simulation box with a minimum distance of 1.2 nm between the protein and the box boundaries. Long-range electrostatic interactions were treated using the Particle Mesh Ewald (PME) method^62^ with a Fourier grid spacing of 0.132 nm and a real-space cutoff of 1.2 nm. All bonds involving hydrogen atoms were constrained using the LINCS algorithm.^63^

For each system, energy minimization was first performed, followed by gradual heating from 50 to 300 K over 100 ps using an annealing protocol. The system was then equilibrated in the NPT ensemble for 1 ns with a 1 fs integration time step, employing the Berendsen thermostat (τ_*T*_ = 0.1 ps) and barostat^64^ (τ_*P*_ = 1.0 ps) at 300 K and 1.30 bar, corresponding to the average pressure of liquid water under the chosen model. Subsequently, a 10 ns NPT simulation was carried out using the Nosé–Hoover thermostat^65^ (τ_*T*_ = 1.0 ps) and the Parrinello–Rahman barostat^66^ (τ_*P*_ = 2.0 ps) at 300 K and 1.30 bar, with a 2 fs integration step. A representative frame at the correct pressure was extracted from this simulation and used to initiate the production run. Two sets of production MD simulations of 100 ns each were then performed in the NVT ensemble at 300 K using the Nosé–Hoover thermostat^65^ (τ_*T*_ = 1.0 ps) and a 2 fs integration step. Coordinates were saved every 1 ps.

## Supplementary Material


