# Supplementary material for: A Single Active-Site Water Molecule Encodes Two Distinct Proton-Coupled Electron Transfer Pathways in Fatty Acid Photodecarboxylase
Source: ACS Omega. 2026 Oct 21;11(43):66663–73. doi: 10.1021/acsomega.6c06141 (PMC13737892; doi:10.1021/acsomega.6c06141)
Supplement: Supplementary file 1 [file ao6c06141_si_001.pdf]

Supporting Information: A Single Active-Site Water Molecule Encodes Two Distinct Proton-Coupled Electron Transfer Pathways in Fatty Acid Photodecarboxylase

Gianluca Dell'Orletta,[*,†] Dongping Zhong,[‡] Massimiliano Aschi,[†] and Isabella Daidone[*,†]

†*Department of Physical and Chemical Sciences, University of L'Aquila, via Vetoio (Coppito 1), 67100 L'Aquila, Italy*

‡*Center for Ultrafast Science and Technology, School of Chemistry and Chemical Engineering, Shanghai Jiao Tong University, Shanghai, 200240, China*

E-mail: gianluca.dellorletta@graduate.univaq.it; isabella.daidone@univaq.it

Contents

1 Figures and Table **S-3**

1 Figures and Table

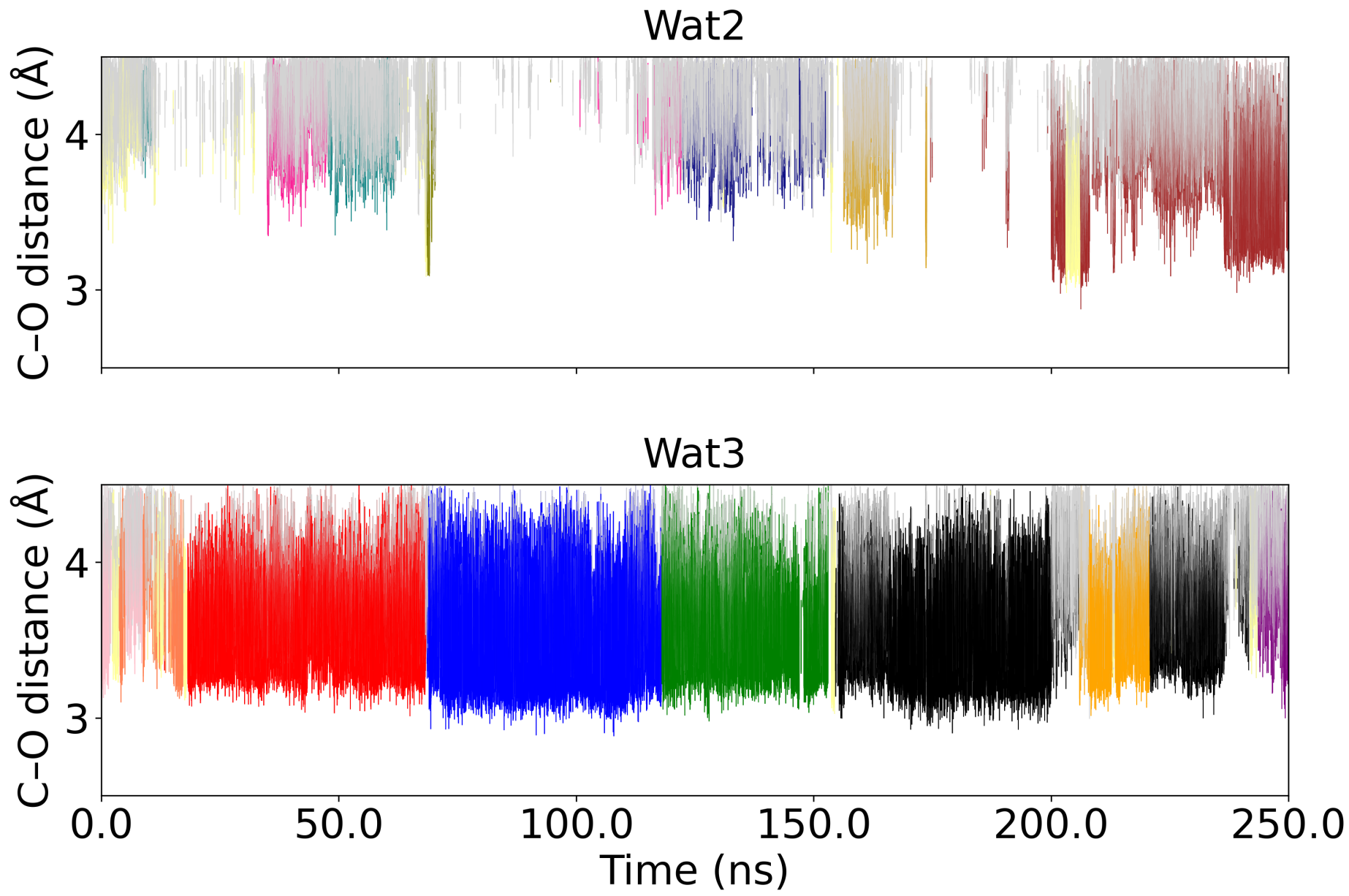

Figure S1: Time evolution of the distance between the C_α atom of R-COO^- and the oxygen atom of Wat2 (top panel) and Wat3 (lower panel). Only water molecules within 4.5 Åof $C\alpha$ are considered to ensure proximity to the reactive $C\alpha$ position. Different colors indicate distinct water molecules occupying the Wat2 and Wat3 sites. Gray indicates frames in which the corresponding water molecule is not the closest water molecule to C_α. The Wat2 and Wat3 sites are simultaneously occupied in approximately 31% of the molecular dynamics trajectory frames.

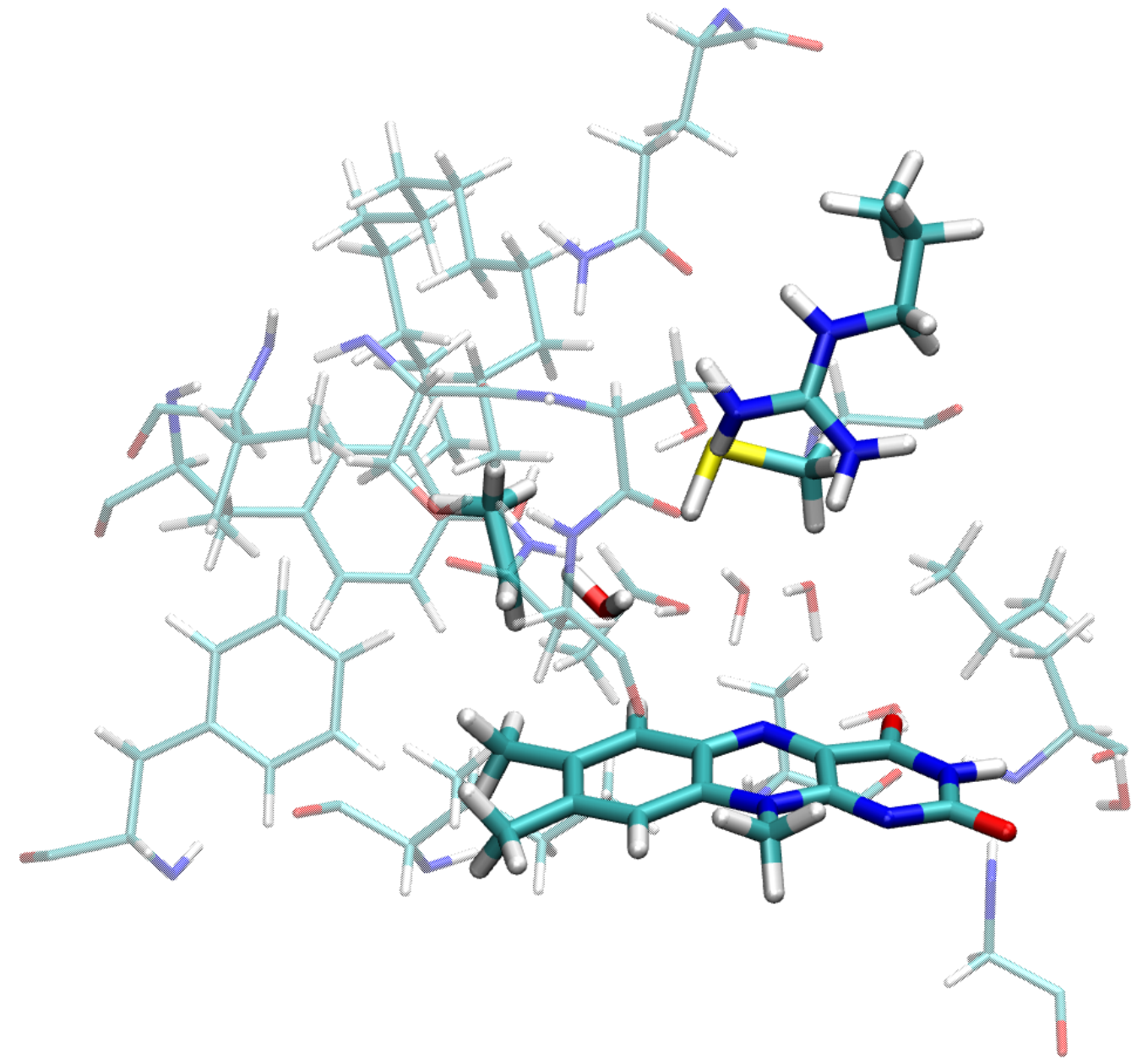

Figure S2: Representation of the QM region (opaque) and the MM active region (transparent) used for the PCET scan calculations for the C432-in basin.

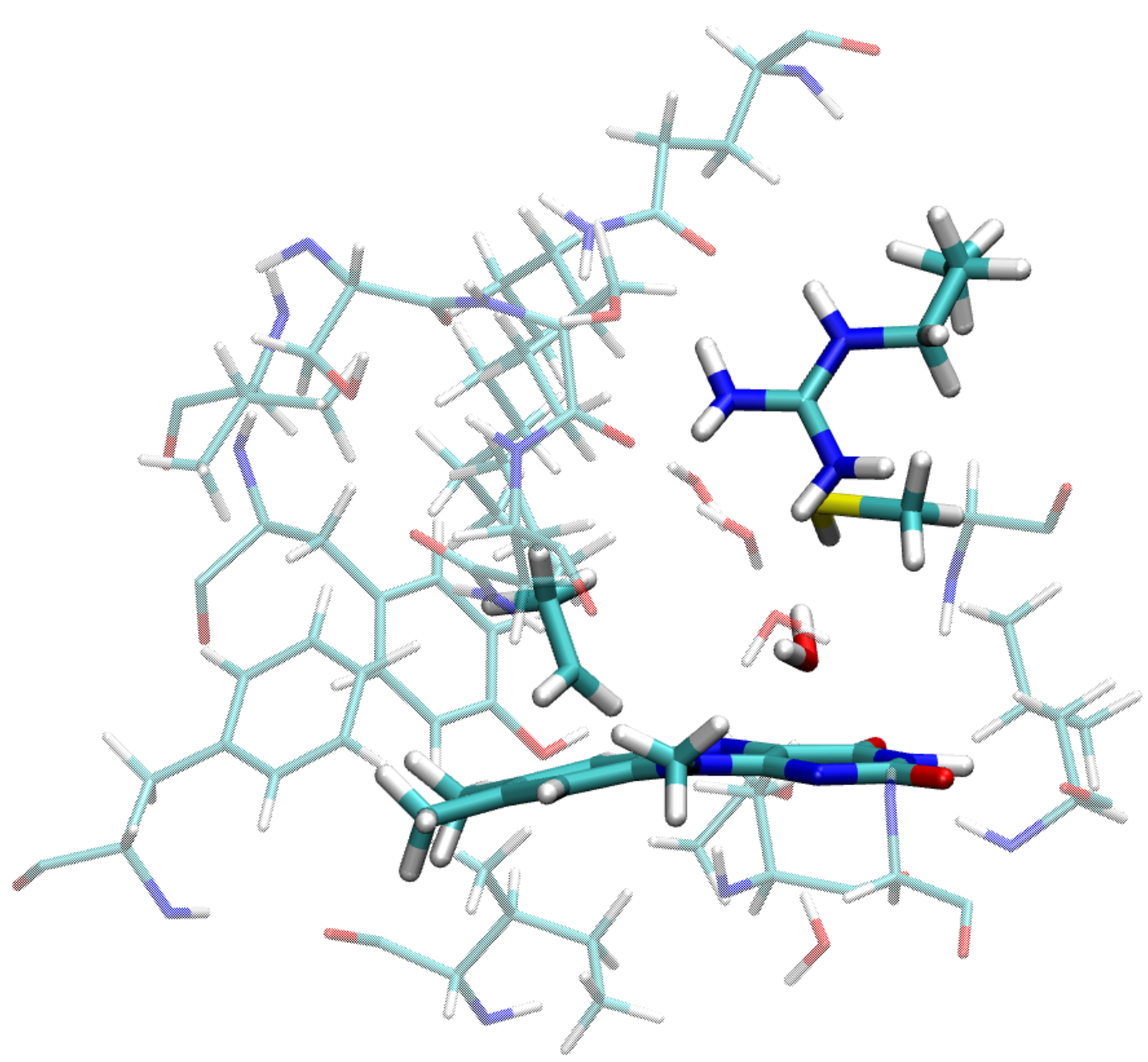

Figure S3: Representation of the QM region (opaque) and the MM active region (transparent) used for the PCET scan calculations for the C432-out basin.

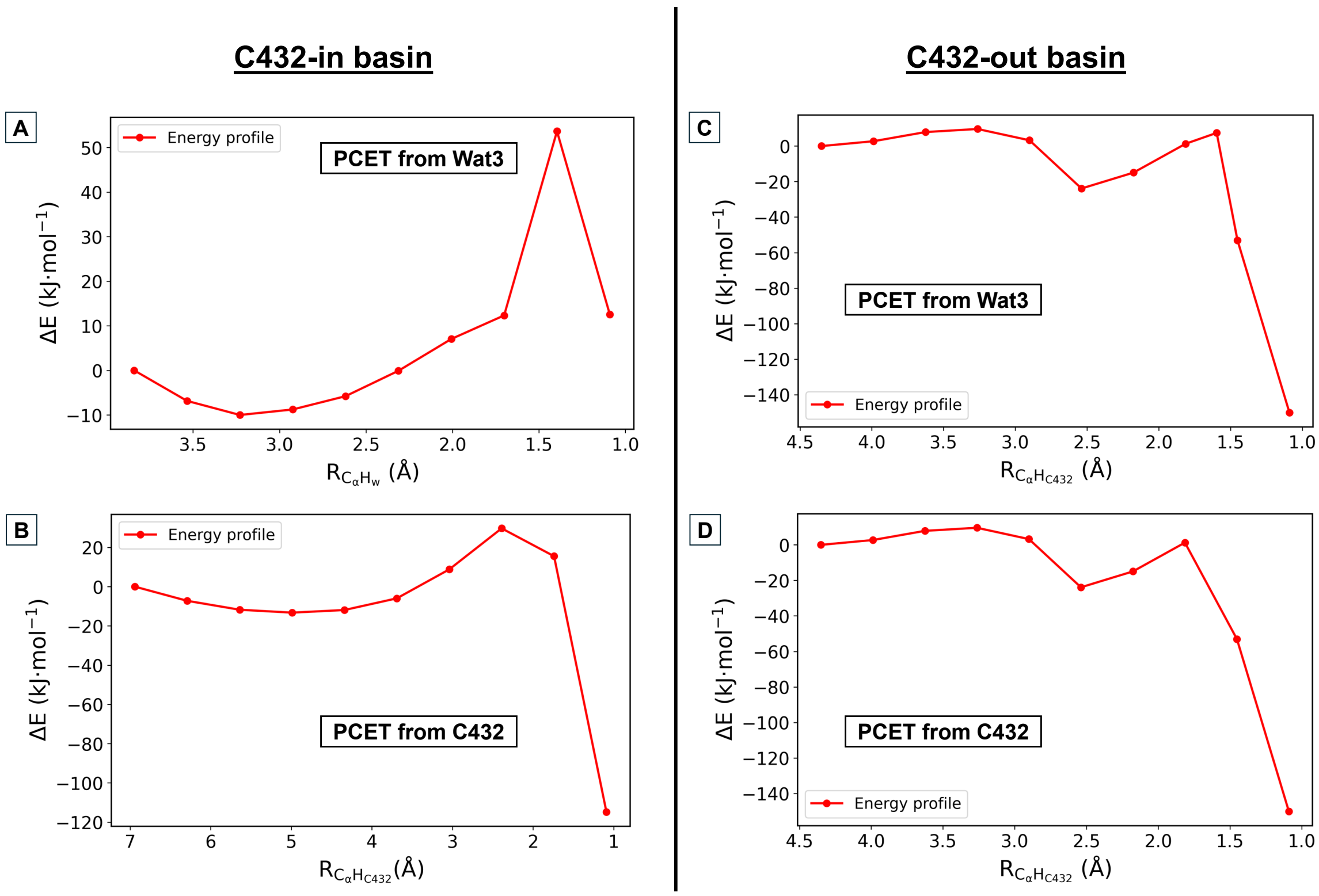

Figure S4: QM/MM energy profiles for the PCET reaction from different proton donors and conformational basins. Energy profiles are reported as ΔE (kJ·mol^{-1}) relative to the reactant state. A) QM/MM energy profile for PCET from Wat3 for a representative structure of the C432-out basin. B) QM/MM energy profile for PCET from C432 for a representative structure of the C432-out basin. C) QM/MM energy profile for PCET from Wat3 for a representative structure of the C432-in basin. D) QM/MM energy profile for PCET from C432 for a representative structure of the C432-in basin.

Table S1: Distances and angles used to characterize (potential) hydrogen bonds in the reactant, transition state, and product for the water-relay PCET and direct PCET mechanisms along scans starting from the C432-in basin.

**Water-relay PCET**			
	Reactant	Transition state	Product
$R_{O_{T430}O_{Wat3}}$ (Å)	2.75	2.82	2.66
$H_{T430}O_{T430}\cdots O_{Wat3}$	7.31	11.12	5.12
$R_{S_{C432}O_{Wat3}}$ (Å)	3.67	3.62	3.23
$H_{C432}S_{C432}\cdots O_{Wat3}$	16.01	18.61	6.19
$H_{C432}O_{Wat3}\cdots S_{C432}$	8.95	10.58	14.33
$R_{O_{Wat3}O_{Y466}}$ (Å)	2.71	2.82	2.82
$H_{Wat3}O_{Wat3}\cdots O_{Y466}$	8.96	2.59	11.03
$R_{O_{Wat3}O_{CO_2}}$ (Å)	2.91	3.16	2.90
$H_{Wat3}O_{Wat3}\cdots O_{CO_2}$	30.18	71.36	56.88
**PCET**			
	Reactant	Transition state	Product
$R_{O_{T430}O_{Wat3}}$ (Å)	2.70	2.69	2.67
$H_{T430}O_{T430}\cdots O_{Wat3}$	4.82	7.64	4.99
$R_{S_{C432}O_{Wat3}}$ (Å)	3.75	3.35	3.22
$H_{C432}S_{C432}\cdots O_{Wat3}$	16.86	73.12	61.43
$H_{Wat3}O_{Wat3}\cdots S_{C432}$	68.55	16.62	16.92
$R_{O_{Wat3}O_{Y466}}$ (Å)	2.74	2.74	2.77
$H_{Wat3}O_{Wat3}\cdots O_{Y466}$	13.66	7.49	6.83
$R_{O_{Wat3}O_{CO_2}}$ (Å)	2.80	3.18	2.95
$H_{Wat3}O_{Wat3}\cdots O_{CO_2}$	11.31	55.58	47.51

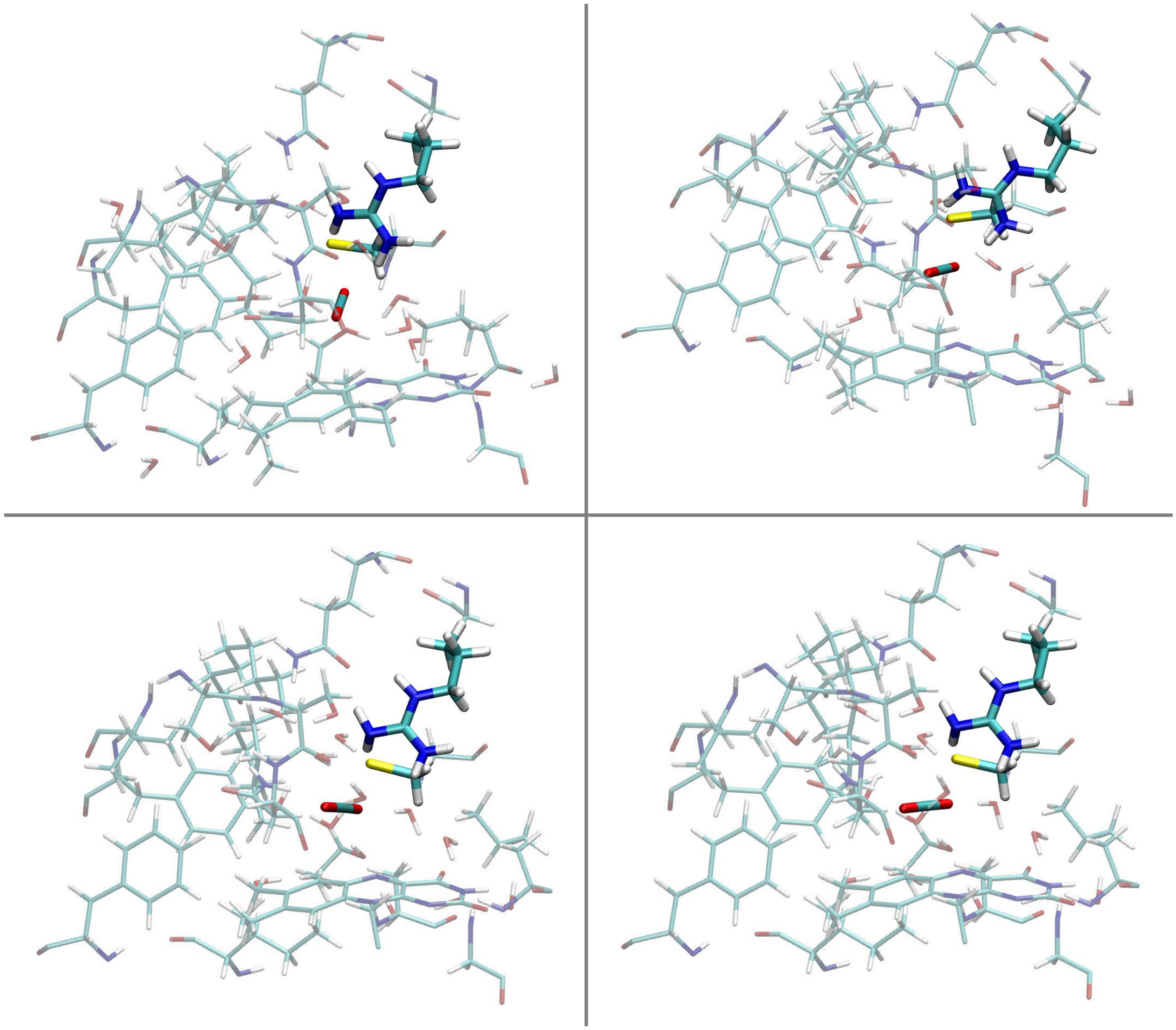

Figure S5: Representation of the QM region (opaque) and the MM active region (transparent) in the starting structures used for the QM/MM scans of thiocarbonate formation.
